# RNA Modifications in Genomic RNA of Influenza A Virus and the Relationship between RNA Modifications and Viral Infection

**DOI:** 10.3390/ijms22179127

**Published:** 2021-08-24

**Authors:** Yuki Furuse

**Affiliations:** 1 Institute for Frontier Life and Medical Sciences, Kyoto University, Kyoto 606-8507, Japan; furusey.kyoto@gmail.com; 2 Hakubi Center for Advanced Research, Kyoto University, Kyoto 606-8501, Japan

**Keywords:** influenza virus, RNA modification, epitranscriptome, host–virus interaction, host factor

## Abstract

Recent studies about the transcriptome-wide presence of RNA modifications have revealed their importance in many cellular functions. Nevertheless, information about RNA modifications in viral RNA is scarce, especially for negative-strand RNA viruses. Here we provide a catalog of RNA modifications including m1A, ac4C, m7G, inosine, and pseudouridine on RNA derived from an influenza A virus infected into A549 cells, as studied by RNA immunoprecipitation followed by deep-sequencing. Possible regions with RNA modifications were found in the negative-strand segments of viral genomic RNA. In addition, our analyses of previously published data revealed that the expression levels of the host factors for RNA modifications were affected by an infection with influenza A virus, and some of the host factors likely have a proviral effect. RNA modification is a novel aspect of host–virus interactions leading to the discovery of previously unrecognized viral pathogenicity mechanisms and has the potential to aid the development of novel antivirals.

## 1. Introduction

RNA modifications are widely distributed in the tRNA and rRNA of living organisms, including all prokaryotes, archaea, and eukaryotes. While some modifications in mRNA, such as 7-methylguanosine (m7G) in the 5’ cap-structure, were first reported in the 1970s [1], the recent advancement of next-generation sequencing, or deep-sequencing, technology, has enabled us to detect RNA modifications in a transcriptome-wide manner, also referred to as epitranscriptomics [2]. Examples of RNA modifications include but are not limited to N6-methyladenosine (m6A), N1-methyladenosine (m1A), 5-methylcytosine (m5C), and N4-acetylcytidine (ac4C) [3,4,5,6]. RNA modifications can affect RNA stability and interactions of RNA with other molecules related to physiological functions such as metabolism, carcinogenicity, and immune response [7,8,9].

RNA modifications are found in viral transcripts as well [10,11]. m6A modifications of adenovirus transcripts are necessary for efficient splicing [12], and the same modifications in herpes virus transcripts regulate the viral life cycle [13]. m6A modifications in transcripts of *Flaviviridae* viruses, including dengue virus, zika virus, and the hepatitis C virus, regulate RNA stability and viral replication [14,15]. m5C is reported to regulate the translation efficiency of mRNA of the human immunodeficiency virus [16], and m6A and ac4C in transcripts of the virus increase its RNA stability [17,18].

There are few studies about RNA modifications in negative-strand RNA viruses because poly-A isolated RNA was commonly used to investigate RNA modifications. Poly-A RNA isolation cannot capture non-polyadenylated RNA molecules, including genomic RNA of negative-strand RNA viruses. Interestingly, a previous study described the presence of m6A in the negative-strand RNA of the influenza A virus even though they performed poly-A RNA isolation [19]. Another approach by isolating viral genomic RNA from viral particles found RNA modifications in the genomes of *Pnuemoviridae* viruses [20,21].

Here, we conducted a transcriptome-wide RNA modification analysis of both positive-strand and negative-strand RNA of the influenza A virus in host cells. The negative-strand RNA genome of the influenza A virus consists of eight segments: PB2, PB1, PA, HA, NP, NA, M, and NS. Upon infection, the viral ribonucleoprotein complex is transferred into the cellular nucleus, where the viral RNA polymerase synthesizes two kinds of positive-strand RNA—one is a perfectly complementary sequence of the viral genome for genomic replication, and the other is mRNA with cap-structure at the 5′ end and poly-A tail at the 3´ end for protein translation [22,23]. To our knowledge, the presence of RNA modifications other than m6A in RNA from the virus has not been reported so far [19].

To investigate RNA modifications in both positive-strand and negative-strand RNA from the influenza A virus, we performed RNA immunoprecipitation (RIP) with antibodies against RNA modifications using total RNA samples from virus-infected cells. Although the experiments enriched a large amount of the host’s rRNA, we took advantage of SMART-seq technology that can remove cDNA molecules derived from rRNA during the deep-sequencing sample preparation process [24]. Consequently, we provide a catalog of various RNA modifications in the viral genome. Furthermore, we evaluated a relationship between host factors related to RNA modifications and viral infection by reanalyzing data from published studies.

## 2. Results

### 2.1. Inosine

A549 cells were infected with an influenza A virus (PR8 strain) at MOI 0.1, and intracellular RNA was extracted at 16 h post-infection. The RNA sample was incubated with magnesium ions for fragmentation into pieces of ~100 nucleotides. The fragmented RNA molecules that contained inosine were isolated by RIP using an antibody against the RNA modification, followed by deep-sequencing to detect RIP-enriched regions. A RIP-enriched region was defined as a region enriched at least two-fold in two biological duplicate experiments.

**Figure 1:**
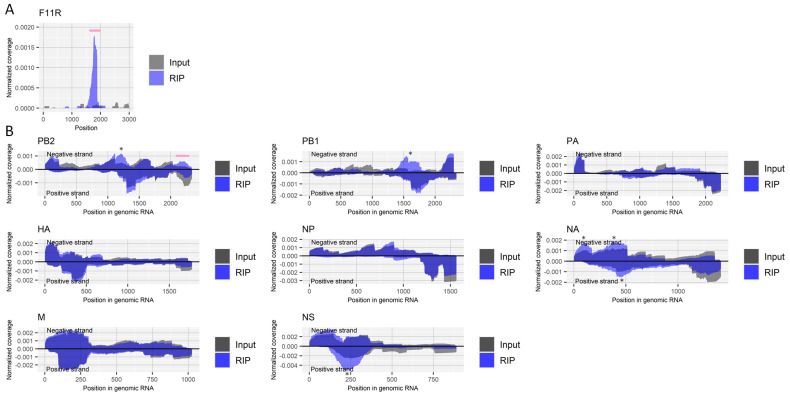
Detection of regions that include inosine in RNA derived from influenza A virus. (**A**) Read coverage of deep-sequencing in the host’s *F11R* gene normalized by the number of read counts mapped to the transcript for RIP and input samples. A pink bar indicates a known region with inosine. (**B**) Read coverage of deep-sequencing in negative-strand RNA (positive values) and positive-strand RNA (negative values) of each viral segment normalized by the number of read counts mapped to each transcript for RIP and input samples. A pink bar indicates an enriched region that possibly contains inosine. The criteria to identify RIP-enriched regions were at least two-fold enrichment, inosine antibody-specific enrichment, and enrichment in two biological duplicates. * non-specifically enriched region that was also observed in other experiments. Results of the biological replicate experiment are available in Supplementary Figure S1.

The enrichment of a region near nucleotide positions 1740–1790 in the host’s *F11R* gene, which is reported to contain inosine [25], validated our experimental procedure (Figure 1A). There were clear peaks at approximately nucleotide position 1200 and position 1600 on the negative-strand RNA of the PB2 and PB1 segments derived from the influenza A virus, respectively (Figure 1B). However, it is likely that this was caused by nonspecific binding of those regions to antibodies, magnetic beads, or other reagents or materials; we repeatedly observed the enrichment of the regions in other experiments using different antibodies. We also conducted a control experiment in which we did not add any antibody for RIP. However, we could not retrieve sufficient RNA in that experiment to perform deep-sequencing. Still, ~two-fold moderate but consistent in duplicate experiments, antibody against inosine-specific enrichment by RIP was found in the negative-strand RNA of PB2 segment (approximately nucleotide position 2200, Figure 1B).

We also investigated the effect of influenza A virus infection on host factors that generate inosine modification, using published transcriptome data from six experiments (Table 1). Viral infection upregulated the expression level of an inosine writer, ADAR (also called ADAR1), observed in five out of six transcriptome experiments, while viral infection downregulated another inosine writer, ADARB1 (also called ADAR2), reported in one out of six experiments (Table 1). We finally investigated the importance of those host factors in the growth of the influenza A virus using the data of eight studies that had performed a screening assay to identify proviral host factors, the knockout/knockdown of which negatively affected viral growth. Yet, viral growth was not disturbed by the knockout/knockdown of ADAR or ADARB1 (Table 1).

### 2.2. N1-Methyladenosine (m1A)

**Figure 2:**
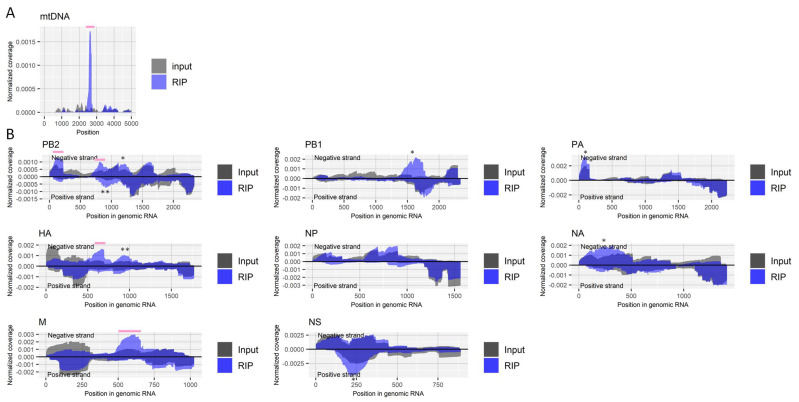
Detection of regions that include m1A in RNA derived from influenza A virus. (**A**) Read coverage of deep-sequencing in the host’s mitochondrial chromosome normalized by the number of read counts mapped to the entire chromosome for RIP and input samples. A pink bar indicates a known region that includes an m1A site. (**B**) Read coverage of deep-sequencing in negative-strand RNA (positive values) and positive-strand RNA (negative values) of each viral segment normalized by the number of read counts mapped to each transcript for RIP and input samples. Pink bars indicate enriched regions that possibly contain m1A. The criteria to identify RIP-enriched regions were at least two-fold enrichment, m1A antibody-specific enrichment, and enrichment in two biological duplicates. * non-specifically enriched region that was also observed in other experiments. ** non-specifically enriched region that was not observed in the biological replicate experiment. Results of the biological replicate experiment are available in Supplementary Figure S2.

Fragmented RNA from influenza A virus-infected cells, as described above, was enriched by RIP using an antibody for m1A followed by deep-sequencing. We validated the RIP experiments by checking the enrichment of the host’s mitochondrial *RNR2* transcript (Figure 2A), which is reported to have a site that undergoes m1A modification (nucleotide position 2617) [32]. The enrichment was observed in several regions of negative-strand viral RNAs, including PB2, HA, and M segments, although some were about two-fold moderately increased but specifically for the antibody in both duplicate experiments (Figure 2B).

Host factors involved with m1A modification, writers [(RRP8 (also called NML), TRMT6, TRMT10C, TRMT61A, and TRMT61B] and erasers (ALKBH1 and ALKBH3), have been identified in previous studies [33]. Upregulation of one writer, TRMT10C, and downregulation of another, RRP8, by influenza A virus infection was found in one of six transcriptome studies, each (Table 1). Changes in the transcription level of the m1A erasers by viral infection were not observed. An m1A writer, TRMT61A, may possess a proviral effect, as suggested by two independent studies that showed the knockdown of that host factor reduced viral titer (Table 1).

### 2.3. Pseudouridine

**Figure 3:**
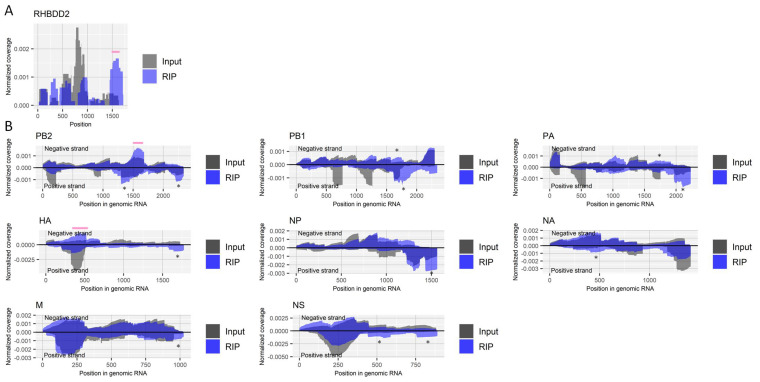
Detection of regions that include pseudouridine in RNA derived from influenza A virus. (**A**) Read coverage of deep-sequencing in the host’s *RHBDD2* gene normalized by the number of read counts mapped to the transcript for RIP and input samples. A pink bar indicates a region known to include a site modified with pseudouridine. (**B**) Read coverage of deep-sequencing in negative-strand RNA (positive values) and positive-strand RNA (negative values) of each viral segment normalized by the number of read counts mapped to each transcript for RIP and input samples. Pink bars indicate enriched regions that possibly contain pseudouridine. The criteria to identify RIP-enriched regions were at least two-fold enrichment, pseudouridine antibody-specific enrichment, and enrichment in two biological duplicates. *, non-specifically enriched region that was also observed in other experiments. Results of the biological replicate experiment are available in Supplementary Figure S3.

We performed RIP with fragmented RNA samples from virus-infected cells using an antibody for pseudouridine. We confirmed the enrichment of a region that contains a known site with pseudouridine (nucleotide position 1580) in the host’s *RHBDD2* gene [34] (Figure 3A). At least two-fold enrichments specific for the antibody were observed in a region near nucleotide position 1600 of the negative-strand PB2 segment and a region near nucleotide position 400 of the negative-strand HA segment in both duplicate experiments (Figure 3B).

Viral infection affected host factors that regulate pseudouridine modification, downregulating DKC1 (also called Cbf5), PUS1, PUS3, and RPUSD2 (also called PUS9), as shown in one of six transcriptome experiments, each (Table 1). Viral infection upregulated another host factor for pseudouridine, NOP10, observed in one of six experiments. Knockout/knockdown screening studies did not identify any of the pseudouridine regulators as a proviral host factor. PUS7 was found to interact with the viral neuraminidase protein (Table 1).

### 2.4. N4-Acetylcytidine (ac4C)

**Figure 4:**
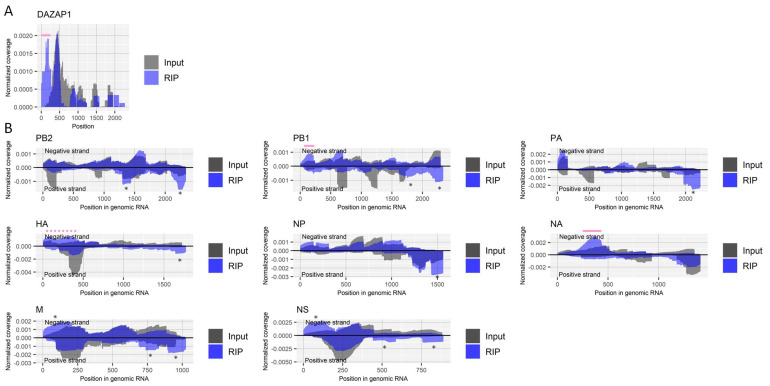
Detection of regions that include ac4C in RNA derived from influenza A virus. (**A**) Read coverage of deep-sequencing in the host’s *DAZAP1* gene normalized by the number of read counts mapped to the transcript for RIP and input samples. A pink bar indicates a previously reported region with ac4C. (**B**) Read coverage of deep-sequencing in negative-strand RNA (positive values) and positive-strand RNA (negative values) of each viral segment normalized by the number of read counts mapped to each transcript for RIP and input samples. Solid and dashed pink bars indicate sharply and moderately enriched regions that possibly contain ac4C, respectively. The criteria to identify RIP-enriched regions were at least two-fold enrichment, ac4C antibody-specific enrichment, and enrichment in two biological duplicates. * non-specifically enriched region that was also observed in other experiments. Results of the biological replicate experiment are available in Supplementary Figure S4.

We further performed RIP using a recently developed antibody for ac4C [3] with fragmented RNA samples from virus-infected cells. The enrichment of the 5′ untranslated region (UTR) of the host’s *DAZAP1* gene by RIP was reported in a previously published epitranscriptome study for ac4C [3] and successfully observed in our results as well (Figure 4A). Enrichments were observed in several regions of RNA derived from the influenza A virus (Figure 4B). While many enrichments seemed nonspecific, as seen in other experiments in the present study, a region near nucleotide position 150 in the negative strand of the PB1 segment and a region near nucleotide position 350 in the negative strand of the NA segment were specifically enriched by the antibody against ac4C at least two-fold in both duplicate experiments. Relatively broad enrichment was also observed for approximately 400 bases at the 5′ end of the negative strand of the HA segment, which may suggest multiple ac4C sites around the region.

NAT10 is a host factor responsible for the regulation of ac4C modification [3]. NAT10 has physical interactions with viral proteins, including PB1, NP, NA, and M1 (Table 1). Of six transcriptome studies, one found downregulation of NAT10 in human cells infected with the influenza A virus. Knockdown of the host factor negatively regulated viral growth, suggesting its proviral role (Table 1).

### 2.5. 7-Methylguanosine (m7G)

**Figure 5:**
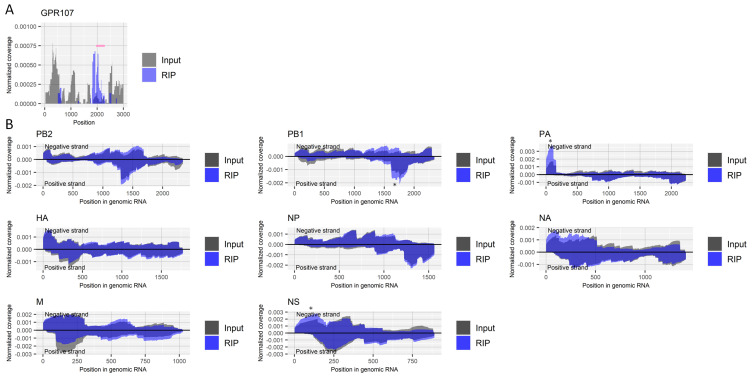
Search for regions that include m7G in RNA derived from influenza A virus. (**A**) Read coverage of deep-sequencing in the host’s *GPR107* gene normalized by the number of read counts mapped to the transcript for RIP and input samples. A pink bar indicates a region with a possible m7G site. (**B**) Read coverage of deep-sequencing in negative-strand RNA (positive values) and positive-strand RNA (negative values) of each viral segment normalized by the number of read counts mapped to each transcript for RIP and input samples. *, non-specifically enriched region that was also observed in other experiments. Results of the biological replicate experiment are available in Supplementary Figure S5.

We next investigated the presence of m7G modification in RNA from the influenza A virus. Because the RNA modification is abundant at the cap-structure of the 5′ end of both host and viral mRNA, we first treated RNA samples with RNA 5′ pyrophosphohydrolase to remove the cap-structure, followed by fragmentation and RIP using an antibody for m7G. Although the presence of m7G in the internal mRNA of eukaryotic cells is controversial [35,36], our RIP experiment enriched a region near the site within the 3´ UTR of the host’s *GPR107* gene (nucleotide position 2051), which was reported by Zhang et al. to possess the RNA modification [35] (Figure 5A). As for RNA derived from the influenza A virus, we detected no specifically enriched region by the RIP (Figure 5B).

One type of m7G writer, WDR4, was downregulated after viral infection, as shown in two of six experiments (Table 1). Knockout/knockdown experiments found no effect on viral growth by host factors responsible for m7G.

### 2.6. N6-Methyladenosine (m6A) and 5-Methylcytosine (m5C)

We also investigated host–virus interaction through m6A and m5C RNA modifications analyzing data from previously published studies, although we could not perform RIP experiments for the modifications due to technical and logistic issues. m6A and its function have been extensively studied in previous studies; host factors related to the RNA modification have been identified, including writers [METTL3, METTL14, RBM15, VIRMA (also called KIAA1429), WTAP, and ZC3H13]; readers (YTHDC1, YTHDF1, and YTHDF2); and erasers (ALKBH5 and FTO) [37]. Transcriptome analyses showed that infection with influenza A virus upregulated the expression level of WTAP (two of six experiments) but downregulated METTL3, RBM15, and VIRMA (one of six experiments, each) (Table 1). Among m6A readers, YTHDC1 was upregulated by viral infection (three of six experiments), while the expression levels of YTHDF1 and YTHD2 were not affected. Downregulation of one of the m6A erasers, FTO, by viral infection was also reported. Overall, influenza A virus infection seems to cause increased m6A modification and/or the effect thereof.

Knockdown of an m6A writer, WTAP, decreased the viral growth, reported in two independent studies (Table 1). Furthermore, the knockdown of m6A readers YTHDC1, YTHDF1, and YTHDF2 also negatively affected the viral growth, suggesting a proviral effect of m6A modification. Two host factors, YTHDF1 and YTHDF2, were reported to physically interact with viral proteins including PB2 and NP (Table 1).

Among the host factors responsible for m5C modification, infection with influenza A virus downregulated NOP2 (two out of six experiments, also called NSUN1); upregulated NSUN3 (one of six experiments), NSUN6 (one of six experiments), and NSUN7 (three of six experiments but downregulated in one experiment), and did not affect the expression levels of NSUN2, NSUN4, or NSUN5. Changes in the expression levels of TRDMT1 (also called DNMT2), another m5C writer, by viral infection yielded contradictory results: upregulation in one study and downregulation in another. Knockdown of TRDMT1 significantly decreased viral growth, suggesting its proviral role (Table 1). NOP2 and NSUN2 were reported to have physical interactions with a viral protein, NP, as shown by a co-immunoprecipitation experiment (Table 1).

## 3. Discussion

**Figure 6:**
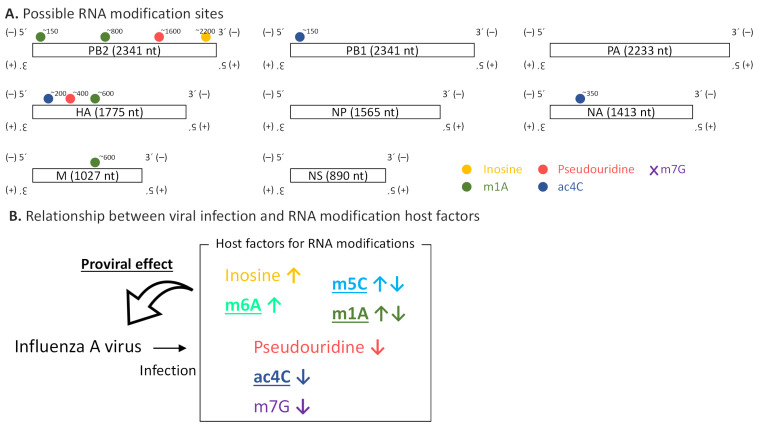
Summary of the relationship between RNA modifications and influenza A virus. (**A**) Catalog of possible RNA modifications and their positions in RNA derived from influenza A virus identified in the present study. m7G sites were not detected. (**B**) Relationship between viral infection and host factors for RNA modification is illustrated. Changes in expression levels (↑upregulation or ↓downregulation) of host factors by viral infection are depicted as vertical arrows next to the names of RNA modifications. Host factors related to RNA modifications described in bold characters with underline possibly have a proviral effect.

In this study, we provide a catalog of possible RNA modifications including inosine, m1A, pseudouridine, and ac4C on the RNA molecules from the influenza A virus (Figure 6A). We documented the genome/transcriptome-wide landscape of RNA modifications in the influenza A virus for the first time, with the exception of m6A [19]. Significantly, the identified possible RNA modifications were observed in negative-strand genomic RNA, which has been rarely investigated from the perspective of RNA modification.

It should be noted that the specificity of the RIP experiment was not adequate to conclude the presence of RNA modifications. It is commonly reported and discussed that there were few overlaps in identified regions that possibly contain RNA modifications among different studies using similar methodologies, even for well-studied model organisms [2,38]. The sensitivity and specificity of omics studies, including the present study, should be further investigated and validated to identify true RNA modification sites. Utilization of photo-crosslinking technology, conjugating RNA modifications and antibodies followed by RNase treatment, could improve the resolution, sensitivity, and possibly specificity of RIP experiments [39]. However, in such experiments, the depletion of rRNA sequences from non-polyadenylated RNA including the viral genome of the influenza virus will be challenging.

Direct RNA sequencing by nanopore sequencing technology will make a significant contribution to detecting RNA modifications [40,41], although its accuracy and applicability to identify various RNA modifications are still developing [42]. Future studies should also validate the possible RNA modification sites by determining whether the RNA modification signals will be lost upon mutation of the sites or inhibition of the host factors responsible for those modifications. Methodologies such as ICE-seq for inosine and ψ-seq for pseudouridine, by which chemical reactions on RNA molecules followed by deep-sequencing can identify specific sites with particular RNA modifications [34,43], should be considered as well for further validation.

Still, the present study suggested a variety of RNA modifications in RNA derived from the negative-strand RNA virus. In this study, A549 cells were infected with the influenza A virus (PR8 strain) at MOI 0.1, and intracellular RNA was extracted at a single time point, 16 h post-infection. Because A549 is a cancer cell line from the human lung, it could be subject to non-native genomic or regulatory disruptions. In the future, we should further explore the findings’ generalizability using different cells such as primary bronchial/tracheal epithelial cells, in vivo models, and clinical samples. It is possible that RNA modifications in the viral genome differ among cell types (for example, between epithelial cells and immune cells) and anatomical and physiological conditions (for example, between the upper respiratory tract and the lower respiratory tract). RNA modifications in the viral genome of viral strains other than PR8 should also be tested in the future. In our experiment, the infections were not synchronized by high MOI because we did not have prior knowledge about the dynamics of RNA modifications in the viral RNA during the viral life cycle. Future studies are expected to understand how and when those RNA modifications in the viral genome are made.

We also showed how viral infection affects the host’s RNA modification factors and vice versa (Figure 6B). Many host factors involved in RNA modifications have proviral roles. RNA modifications on the viral genome could increase its stability [44]. Besides, they might be engaged with viral genomic replication and genome packaging, and they could help evade recognition by the host’s innate immunity [45]. It is also possible that RNA modification host factors regulate viral growth via RNA modifications on not viral RNA but host transcripts [46,47].

Inhibiting RNA modifications can be an interesting option for the treatment of viral infections. That could decrease the stability of viral RNA suppressing viral growth and/or enhance the recognition of viral RNA by innate immunity leading to the enhancement of antiviral response. Furthermore, it is intriguing to figure out how the effect of RNA modifications has influenced the evolution of the viral genome. RNA modification is a novel aspect of host–virus interactions leading to a discovery of previously unrecognized mechanisms of the viral life cycle.

## 4. Materials and Methods

### 4.1. Cell Lines and Virus

A549 cell lines, MDCK cell lines, and influenza A virus [A/PR8/1934 (H1N1)] were obtained from ATCC (Manassas, VA, USA). A549 cells and MDCK cells were maintained in high-glucose DMEM supplemented with 10% fetal bovine serum. Influenza A virus was propagated and titrated using MDCK cells.

### 4.2. Viral Infection and RNA Extraction

Influenza A virus was inoculated into subconfluent A549 cells with 0.25 µg/mL TPCK-treated trypsin at MOI 0.1 plaque-forming unit/cell. Cells were washed with PBS three times, and intracellular RNA was extracted using TRIzol (Thermo Fisher Scientific, Waltham, MA, USA) and Direct-zol (Zymo Research, Irvine, CA, USA) with DNase treatment at 16 h after inoculation. All experiments from viral infection to deep-sequencing were done in biological duplicates (Supplementary Figures S1–S5).

### 4.3. RNA Fragmentation

Forty micrograms of total RNA for the stringent RIP protocol, 80 µg of total RNA for the mild RIP protocol, and 5 µg of total RNA for the m7G-RIP protocol were fragmented using the NEBNext Magnesium RNA Fragmentation Module (NEB, Ipswich, MA, USA) according to the manufacturer’s protocol. Incubation with magnesium ions was performed at 94 °C for 5 min, followed by RNA purification by ethanol precipitation. The peak size of fragmented RNA was ~100 bases, confirmed using Bioanalyzer (Agilent Technologies, Santa Clara, CA, USA).

### 4.4. Removal of Cap-Structure for m7G-RIP

Fragmented RNA was incubated with 500 U/mL RNA 5′ pyrophosphohydrolase (NEB) and Thermopol buffer (NEB) at 37 °C for 2 h, followed by the addition of 10 mM EDTA and incubation at 65 °C for 5 min. Treated RNA was then purified using TRIzol LS (Thermo Fisher Scientific).

### 4.5. Antibodies

Antibodies and their concentrations used for the RIP experiments are described in Supplementary Table S1. Because a widely used antibody for m1A, D345-3 (MBL, Tokyo, Japan), was reported to cross-react with adenosine near the cap-structure at the transcription start site, we used a different antibody, ab208196 (Abcam, Cambridge, UK), which a previous study showed has no such cross-reactivity [32].

### 4.6. Stringent RNA Immunoprecipitation for Inosine, m1A, and m7G

RIP experiments were performed as previously reported with a slight modification [4]. Forty microliters of Dynabeads Protein G (Thermo Fisher Scientific) were washed with IPP buffer [150 mM NaCl, 0.1% NP-40, 10 mM Tris-HCl (pH 7.5)] twice and resuspended with 200 µL IPP buffer. The antibody was added as described in Supplementary Table S1 and incubated, rotating for 1 h at room temperature followed by a wash with IPP buffer twice and resuspended with 200 µL IPP buffer. Then, 40 µg of fragmented RNA (or 5 µg RNA for m7G) was added and rotated at 4 °C for 4 h. Thereafter, beads were washed with IPP buffer twice, with low-salt IPP buffer [50 mM NaCl, 0.1% NP-40, 10 mM Tris-HCl (pH 7.5)] twice, and with high-salt IPP buffer [500 mM NaCl, 0.1% NP-40, 10 mM Tris-HCl (pH 7.5)] twice. After removing the washing buffer, 1 mL TRIzol was directly added to the beads to isolate RNA.

### 4.7. Mild RNA Immunoprecipitation for Pseudouridine and ac4C

Because enough RNA was not retrieved for pseudouridine and ac4C using the abovementioned stringent protocol, we further modified the protocol to perform RIP under mild conditions, introducing the following changes: 80 µg of fragmented RNA was used, incubation of antibody with beads was conducted with rotating for 2 h, and incubation of RNA with antibody-conjugated beads was done with rotating at room temperature for 2 h followed by 4 °C for 16 h. The RNA-bound beads were washed with IPP buffer five times before the isolation of RNA by TRIzol.

### 4.8. Deep-Sequencing Sample Preparation and Sequencing

Deep-sequencing sample preparation was performed using a SMART-Seq Stranded Kit (Takara Bio, Japan) as per the manufacturer’s protocol with a slight modification. We skipped an RNA fragmentation step for already fragmented RNA samples for RIP. Deep-sequencing of the prepared samples was conducted using the NovaSeq 6000 (Illumina, San Diego, CA, USA) with a protocol of 2 × 150 base paired-end runs to produce approximately 100 Gb of data (corresponding to 660 million reads).

### 4.9. Bioinformatics

Adapter sequences and low-quality reads in fastq files were removed using Trim Galore with default settings. Sequences of viral positive-strand segments, negative-strand segments, all human transcripts (GRCh38_latest_rna.fna obtained from the National Center for Biotechnology Information), and the mitochondrial genome (GenBank accession number, NC012920) were included in a single fasta file and used as a reference for mapping. NGS reads were mapped against the reference with forward-strand only option using bowtie2 [48]. Site-by-site coverages were calculated using samtools [49], and the coverage at each site was normalized by the number of read counts mapped to each reference transcript.

The criteria to identify RIP-enriched regions include at least two-fold enrichment, each antibody-specific enrichment, and enrichment in two biological duplicate experiments.

### 4.10. Data of Transcriptome Experiments

Transcriptome data from virus-infected cells were retrieved from the Influenza Research Database [50]. All experiments using human cells infected with human influenza A virus were investigated [GSE89008 (H1N1 and H3N2 viruses in human tracheobronchial epithelial cells), GSE97672 (H1N1 and H3N2 viruses in human monocyte-derived macrophages), GSE37571 (H1N1 virus in Calu-3 cells), and GSE40844 (H3N2 virus in Calu-3 cells)]. The database has the RNA expression levels of the host’s genes that were statistically changed by viral infection. Using the data, we determined whether viral infection altered the gene expression levels of the host factors related to RNA modifications.

### 4.11. Proviral Effect of RNA Modification Host Factors

Published data from six studies that performed siRNA knockdown screening [26,27,28,29,30,31], one study that conducted CRISPR knockout screening [51], and one study that performed homozygous gene perturbation screening [52] to identify host factors the knockout/knockdown of which negatively regulate viral growth were investigated. Data from a study by Watanabe et al., which identified host factors co-immunoprecipitated with viral proteins [26], were also analyzed to find RNA modification host factors that physically interact with viral proteins.

### 4.12. Data Availability

The original data of deep-sequencing are available at the DDBJ BioProject database under accession number PRJDB11331.

## Figures and Tables

**Table 1:** Relationship between viral infection and host factors for RNA modification.

Host Factor	RNA Modification	Function	Expression Level Change by Viral Infection in 6 Transcriptome Studies		Provial Effect in 8 Knockout/Knockdown Screening Studies		Viral Protein with Physical Interaction
			Studies Reporting Upregulation	Studies Reporting Downregulation	Studies Identifying Host Factor	Reference	Reference [26]
ADAR	inosine	writer	5	0	0		n.d.
ADARB1	inosine	writer	0	1	0		n.d.
RRP8	m1A	writer	0	1	0		n.d.
TRMT10C	m1A	writer	1	0	0		n.d.
TRMT61A	m1A	writer	0	0	2	[26,27]	n.d.
TRMT6, TRMT61B	m1A	writer	0	0	0		n.d.
ALKBH1, ALKBH3	m1A	eraser	0	0	0		n.d.
DKC1	pseudouridine	writer	0	1	0		n.d.
NOP10	pseudouridine	writer	1	0	0		n.d.
PUS1	pseudouridine	writer	0	1	0		n.d.
PUS3	pseudouridine	writer	0	1	0		n.d.
PUS7	pseudouridine	writer	0	0	0		NA
RPUSD2	pseudouridine	writer	0	1	0		n.d.
GAR1, NHP2, PUS10, TRUB1	pseudouridine	writer	0	0	0		n.d.
NAT10	ac4C	writer	0	1	1	[26]	PB1, NP, NA, M1
WDR4	m7G	writer	0	2	0		n.d.
BUD23, METTL1	m7G	writer	0	0	0		n.d.
METTL3	m6A	writer	0	1	0		n.d.
RBM15	m6A	writer	0	1	0		n.d.
VIRMA	m6A	writer	0	1	0		n.d.
WTAP	m6A	writer	2	0	2	[28,29]	n.d.
METTL14, ZC3H13	m6A	writer	0	0	0		n.d.
YTHDC1	m6A	reader	3	0	1	[30]	n.d.
YTHDF1	m6A	reader	0	0	1	[26]	PB2, NP, NA
YTHDF2	m6A	reader	0	0	1	[26]	PB2, NP, NA, M1
ALKBH5	m6A	eraser	0	0	0		n.d.
FTO	m6A	eraser	0	1	0		n.d.
NOP2	m5C	writer	0	2	0		NP
NSUN2	m5C	writer	0	0	0		NP, NA
NSUN3	m5C	writer	1	0	0		n.d.
NSUN6	m5C	writer	1	0	0		n.d.
NSUN7	m5C	writer	3	1	0		n.d.
TRDMT1	m5C	writer	1	1	1	[31]	n.d.
NSUN4, NSUN5	m5C	writer	0	0	0		n.d.

n.d., not detected.

## Data Availability

The original data of deep-sequencing are available at the DDBJ BioProject database under accession number PRJDB11331.

## Supplementary Materials

The following are available online at https://www.mdpi.com/article/10.3390/ijms22179127/s1.
