# Supplementary material for: RNA Modifications in Genomic RNA of Influenza A Virus and the Relationship between RNA Modifications and Viral Infection
Source: Int J Mol Sci. 2021 Aug 24;22(17):9127. doi: 10.3390/ijms22179127 (PMC8431438; doi:10.3390/ijms22179127)
Supplement: Supplementary file 1 [file ijms-22-09127-s001.zip › ijms-1345558-supplementary.pdf]

Supplementary material

RNA modification	Manufacture	Catalogue number	Original concentration	Used volume in 200 µL reaction
Inosine	MBL	PM098	Unknown	10 µL
m1A	Abcam	ab208196	0.66 µg/µL	20 µL
Pseudouridine	diagenode	c15200247	1.44 µg/µL	5 µL
ac4C	Abcam	ab252215	0.56 µg/µL	5 µL
m7G	MBL	RN017M	1 µg/µL	10 µL

Supplementary table S1. List of antibodies used for RNA immunoprecipitation

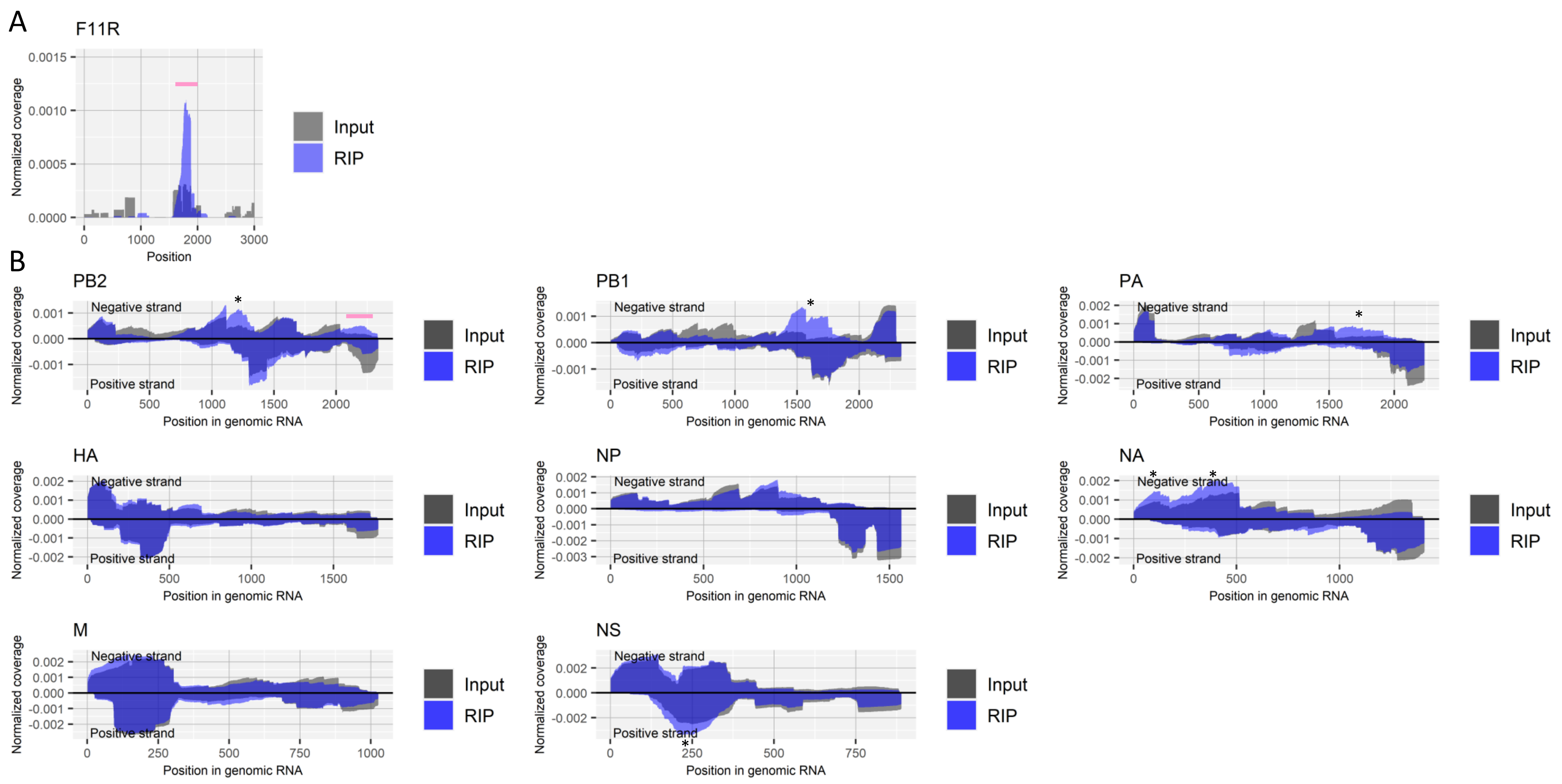

Supplementary figure S1. Detection of regions and sites with inosine in RNA derived from influenza A virus in biological replicate

Results of the biological replicate experiment of Figure 1.

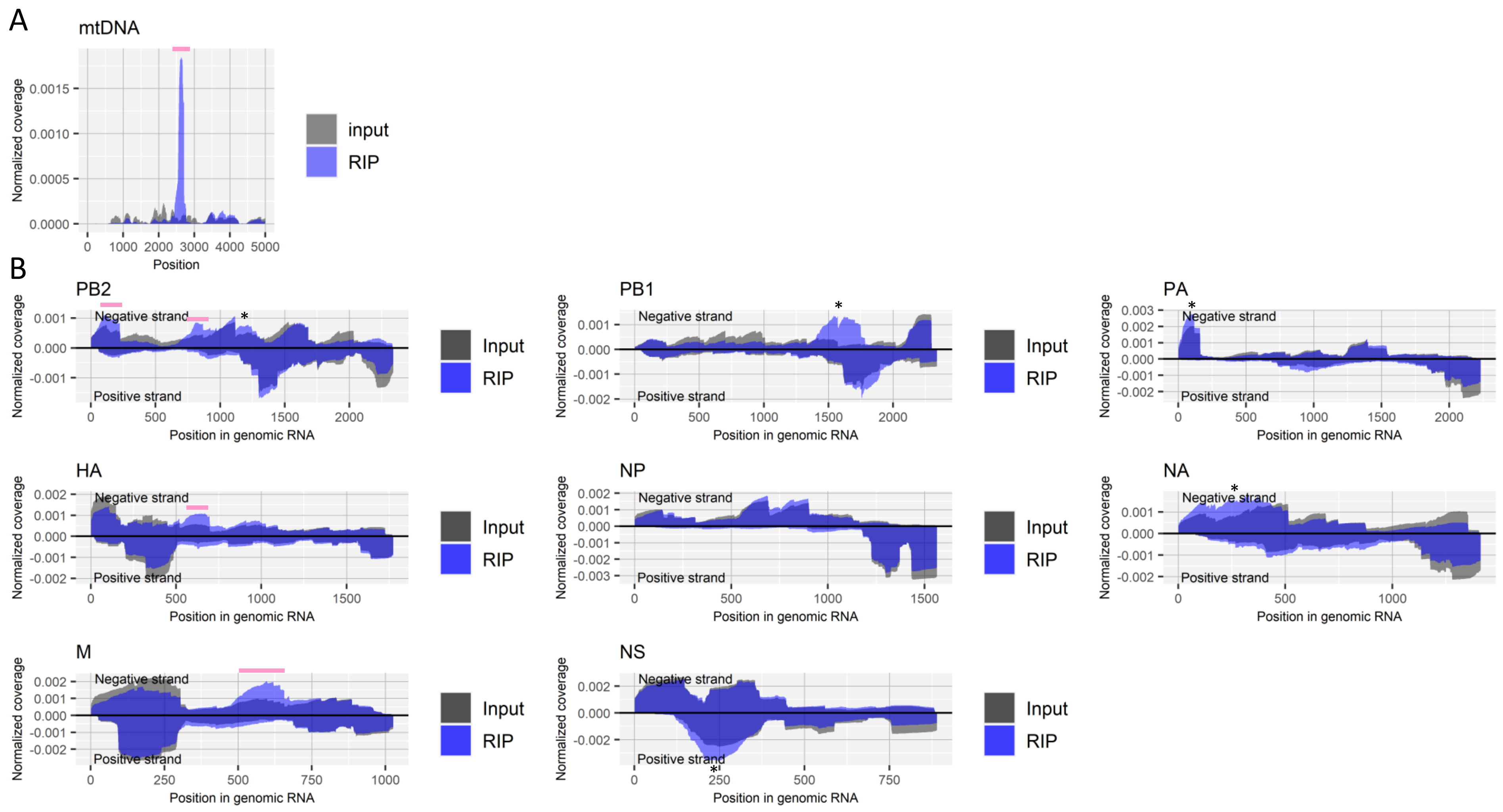

Supplementary figure S2. Detection of regions that include m1A in RNA derived from influenza A virus in biological replicate

Results of the biological replicate experiment of Figure 2.

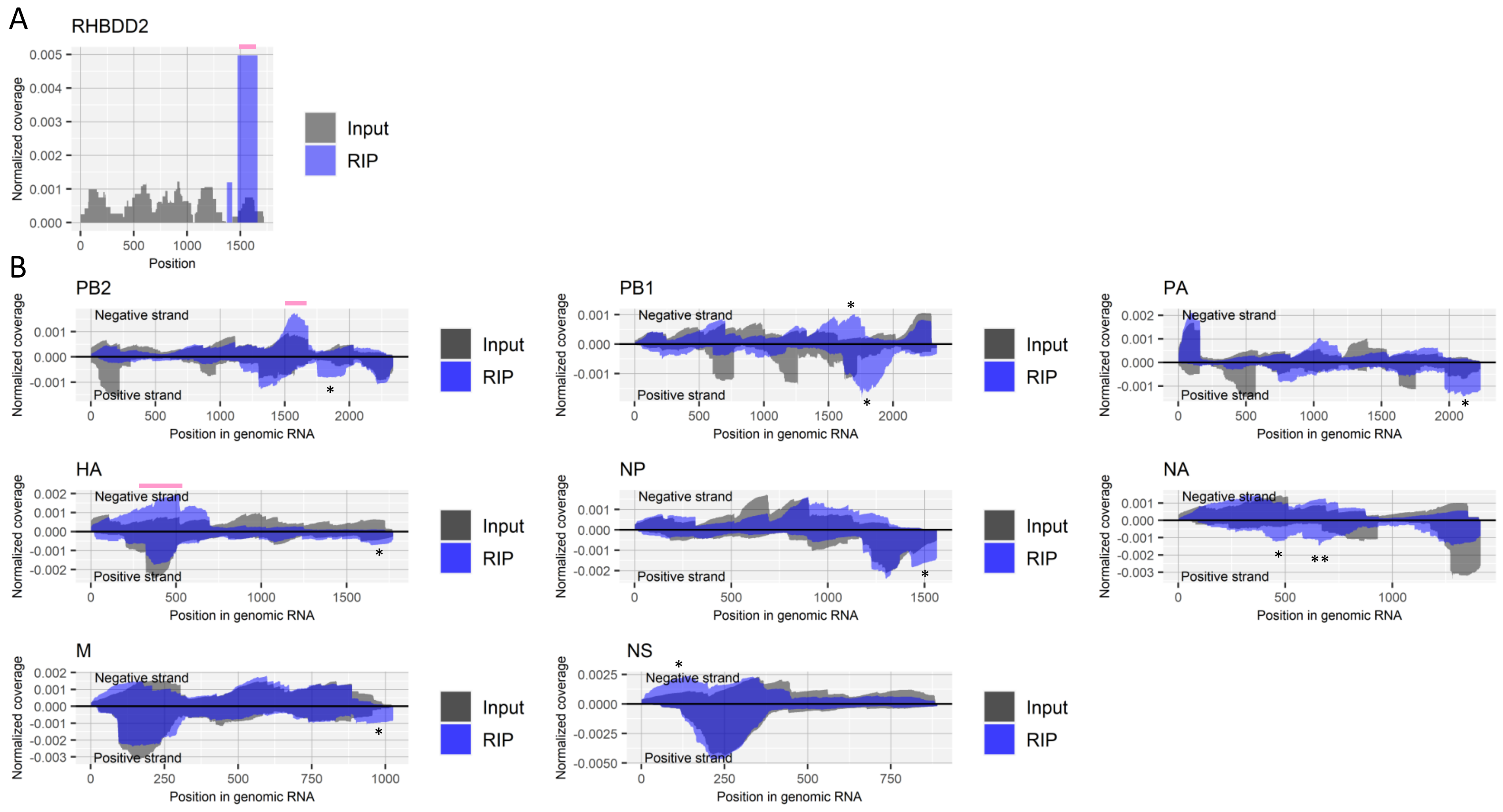

Supplementary figure S3. Detection of regions that include pseudouridine in RNA derived from influenza A virus in biological replicate

Results of the biological replicate experiment of Figure 3.

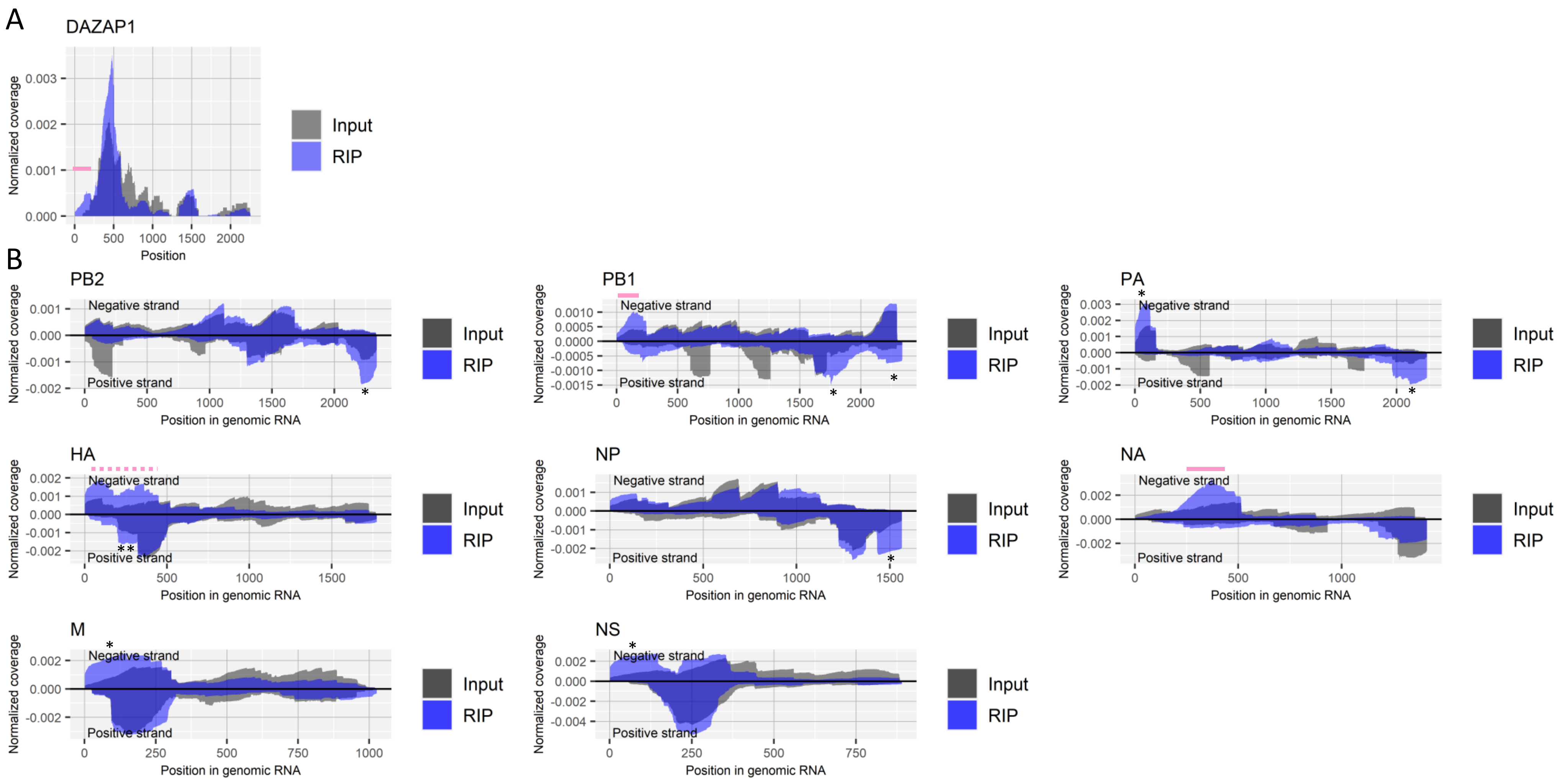

Supplementary figure S4. Detection of regions that include ac4C in RNA derived from influenza A virus in biological replicate

Results of the biological replicate experiment of Figure 4.

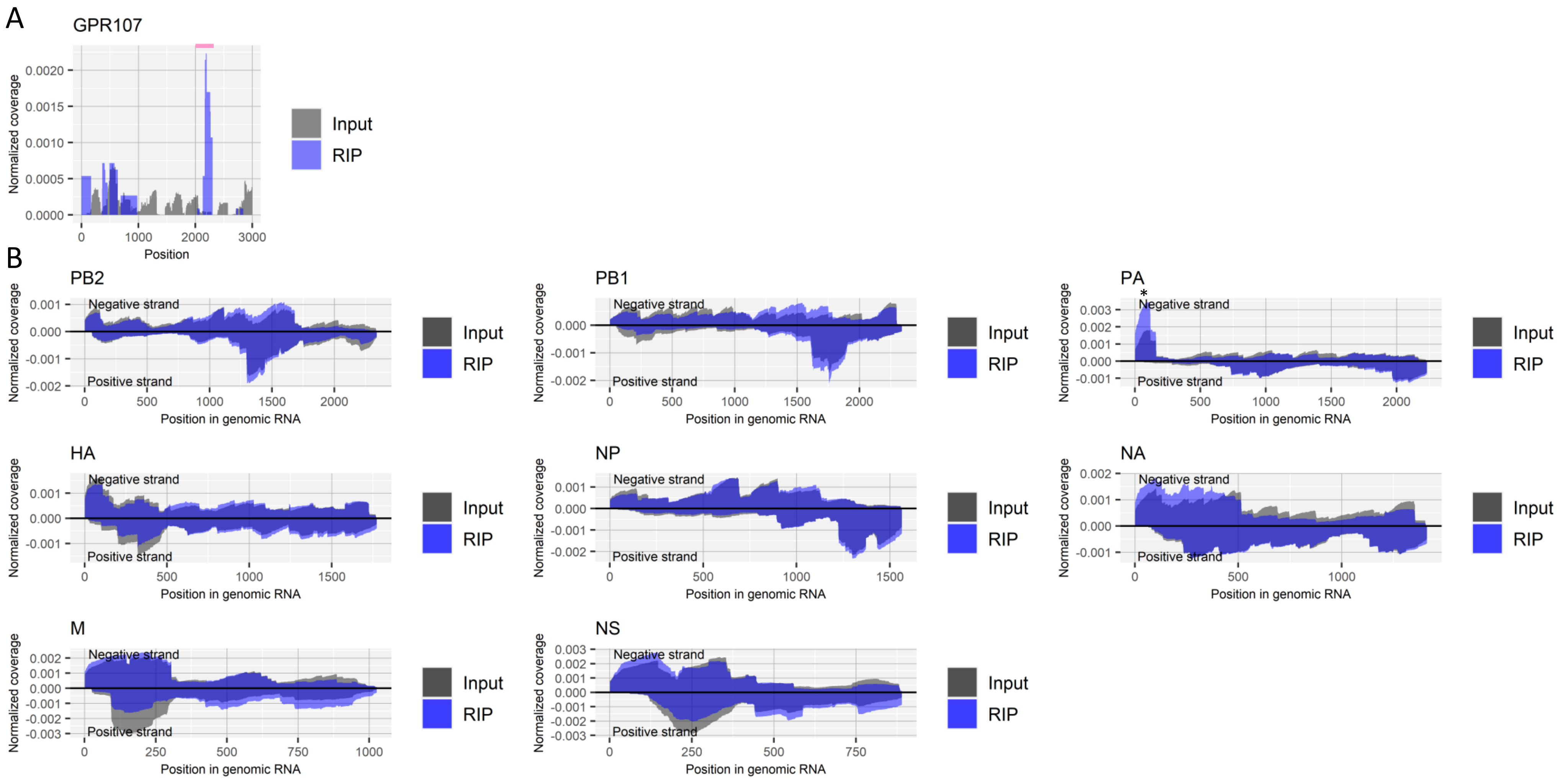

Supplementary figure S5. Search for regions that include m7G in RNA derived from influenza A virus in biological replicate

Results of the biological replicate experiment of Figure 5.
